# Formal Modeling of mTOR Associated Biological Regulatory Network Reveals Novel Therapeutic Strategy for the Treatment of Cancer

**DOI:** 10.3389/fphys.2017.00416

**Published:** 2017-06-13

**Authors:** Zurah Bibi, Jamil Ahmad, Amnah Siddiqa, Rehan Z. Paracha, Tariq Saeed, Amjad Ali, Hussnain Ahmed Janjua, Shakir Ullah, Emna Ben Abdallah, Olivier Roux

**Affiliations:** ^1^Research Centre for Modeling and Simulation, National University of Sciences and TechnologyIslamabad, Pakistan; ^2^Atta-Ur-Rahman School of Applied Biosciences, National University of Sciences and TechnologyIslamabad, Pakistan; ^3^School of Business, Stratford UniversityFalls Church, VA, United States; ^4^IRCCyN UMR Centre National de la Recherche Scientifique 6597, BP 92101Nantes, France

**Keywords:** mTOR signaling pathway, SMBioNet, Biological regulatory networks (BRNs), René Thomas, Qualitative modeling, Model checking, Cancer

## Abstract

Cellular homeostasis is a continuous phenomenon that if compromised can lead to several disorders including cancer. There is a need to understand the dynamics of cellular proliferation to get deeper insights into the prevalence of cancer. Mechanistic Target of Rapamycin (mTOR) is implicated as the central regulator of the metabolic pathway involved in growth whereas its two distinct complexes mTORC1 and mTORC2 perform particular functions in cellular propagation. To date, mTORC1 is a well defined therapeutic target to inhibit uncontrolled cell division, while the role of mTORC2 is not well characterized. Therefore, the current study is designed to understand the signaling dynamics of mTOR and its partner proteins such as PI3K, PTEN, mTORC2, PKB (Akt), mTORC1, and FOXO. For this purpose, a qualitative model of mTOR-associated Biological Regulatory Network (BRN) is constructed to predict its regulatory behaviors which may not be predictable otherwise. The depleted expression of PTEN and FOXO along with the overexpression of PI3K, mTORC2, mTORC1 and Akt is predicted as a stable steady state which is in accordance with their observed expression levels in the progression of various cancers. The qualitative model also predicts the homeostasis of all the entities in the form of qualitative cycles. The significant qualitative (discrete) cycle is identified by analyzing betweenness centralities of the qualitative (discrete) states. This cycle is further refined as a linear hybrid automaton model with the production (activation) and degradation (inhibition) time delays in order to analyze the real-time constraints for its existence. The analysis of the hybrid model provides a formal proof that during homeostasis the inhibition time delay of Akt is less than the inhibition time delay of mTORC2. In conclusion, our observations characterize that in homeostasis Akt is degraded with a faster rate than mTORC2 which suggests that the inhibition of Akt along with the activation of mTORC2 may be a better therapeutic strategy for the treatment of cancer.

## Introduction

Cells need continuous supply of resources that maintain intracellular energy and require nutrient levels contributing to macromolecular biosynthesis and serving as an upstream regulator of cell size and growth rate (Schmelzle and Hall, 2000; Wullschleger et al., 2006; Avruch et al., 2006; Sonntag et al., 2012). Compromised growth homeostasis can lead to several diseases including metabolic disorders, aging and cancer (Zoncu et al., 2011). A serine/threonine protein kinase mTOR, a member of the phosphatidylinositol kinase related kinases (PIKKs) family (Schmelzle and Hall, 2000), acts as a central regulator of homeostasis during growth and starvation (Zoncu et al., 2011). Recent studies have shown that dysregulation in mTOR signaling could lead to cancer and other pathologies (Menon and Manning, 2008). In these studies, abnormally elevated levels of mTOR have been linked with several human cancers including prostate, pancreas, liver, breast, colorectal, urinary tract, and female reproductive organs. On the other hand, due to excess nutrients supply, hyperactivation of mTOR has also been implicated to cause diabetes (Zoncu et al., 2011). Moreover, being the central regulator of growth, mTOR also monitors the process of aging (Zoncu et al., 2011). mTOR functions in the form of two distinct complexes namely mTOR Complex 1 (mTORC1) and 2 (mTORC2) (Wullschleger et al., 2006; Guertin and Sabatini, 2007). These complexes are distinguished by their unique accessory proteins, i.e., raptor and PRAS40 in case of mTORC1 and rictor, Protor and mSin1 in case of mTORC2 (Hara et al., 2002; Kim et al., 2002; Sarbassov et al., 2004). The function of these accessory proteins is to specify their binding with different substrates and regulators (Hara et al., 2002; Kim et al., 2002; Nojima et al., 2003; Schalm et al., 2003; Wullschleger et al., 2005; Sancak et al., 2007; Pearce et al., 2007). Both mTORC1 and mTORC2 share also some components including mLST8 and Deptor that act as positive and negative regulators, respectively (Loewith et al., 2002).

### Signaling of mTOR

**Figure 1:**
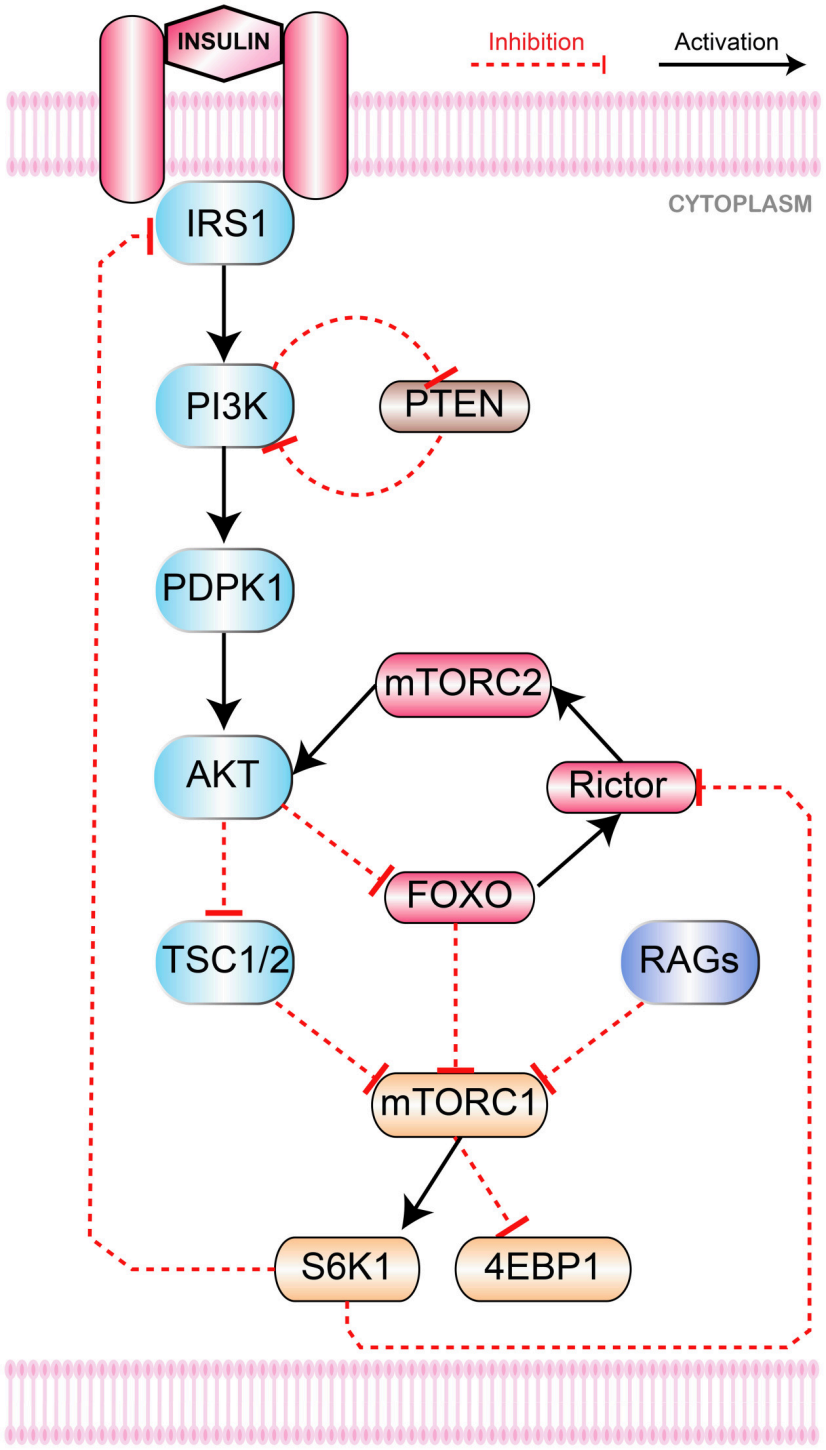
mTOR Signaling Pathway. Collectively growth factors and amino acids (via Rags mediated binding of mTORC1 to Rheb) activate mTORC1. Insulin binds to its IRS1 that activates PI3K and recruits PDPK1 from cell membrane via to PIP3 conversion. PI3K and mTORC2 phoshorylate Akt at Thr308 and Ser473, respectively. Through inhibition of TSC1-TSC2 and FOXO, Akt stimulates mTORC1 to promote mRNA translation and inhibits apoptosis by phosphorylating S6K1 and 4E-BP1. Negative feedback inhibition of IRS1 is initiated by S6K1 to downregulate glucose metabolism. PTEN regulates mutagenic stimulation of PI3K (via PIP3 to PIP2 reconversion) to keep cellular propagation within controlled levels while FOXO is responsible to control hyperactivation of mTORC1.

The mTOR signaling pathway (Figure 1) is initiated by insulin, insulin-like growth factor 1 (IGF1) and Ras (Laplante and Sabatini, 2012) along with others. The binding of insulin with insulin/IGF1 signaling (IIS) receptors causes its autophosphorylation with subsequent recruitment of insulin receptor substrate (IRS) with its cytosolic domain. Activated IRS then activates several downstream effector proteins including PI3K.

The role of PI3K pathway in metabolism and growth is well-established and its dysregulation could result in certain metabolic disorders and cancers (Carracedo and Pandolfi, 2008). In addition to the activation of JNK pathway (Vivanco et al., 2007) that down-regulates PTEN transcription and promotes cellular proliferation by hindering apoptosis, it is also involved in the activation of Akt (Carracedo and Pandolfi, 2008). PI3K phosphorylates and converts phosphatidylinositol (4,5)-bisphosphate (PIP2) to phosphatidylinositol (3,4,5)-trisphosphate to (PIP3) (Engelman et al., 2006; Manning and Cantley, 2007). This event is important for the activation of phosphatidylinositol dependent protein kinase 1 (PDPK1) that eventually stimulates Akt. The activation of Akt is achieved through phosphorylation at two sites i.e., Thr308 and Ser473. Activated PDPK1 phosphorylates Akt at position Thr308 whereas another protein mTORC2 phosphorylates it at Ser473 (Alessi et al., 1997; Sarbassov et al., 2005). Both these phosphorylation events are essential for the complete activation of Akt. Thobe et. al. examined the influence of PI3K pathway kinases on mTORC2 and found PI3K mediated regulation essential for mTORC2 recruitment and further activation Thobe et al. (2017).

Akt regulates several downstream proteins such as Tuberous Sclerosis proteins 1 and 2 (TSC1/TSC2) and FOXO. The heterodimer of TSC1 (hamartin) and TSC2 (tuberin) primarily inhibits the activity of mTORC1 via conversion of active Ras homolog enriched in brain (Rheb)-GAP into inactive Guanosine diphosphate (GDP)-bound Rheb (Inoki et al., 2003; Tee et al., 2003). Akt phoshorylates and inhibits TSC1/TSC2 in order to activate mTORC1 (Inoki et al., 2002; Manning et al., 2002; Potter et al., 2002; Roux et al., 2004; Ma et al., 2005). Akt also elevates the expression of mTORC1 indirectly through the inhibition of FOXO (Guertin et al., 2006; Chen et al., 2010; Zoncu et al., 2011). FOXO acts a homeostatic regulator of cellular energy production and consumption processes under energy stress conditions. Another role of FOXO is to increase the activation of Rictor (a major component of mTORC2) and subsequently mTORC1.

PI3K signaling is mainly buffered through PTEN (Carracedo et al., 2008). PTEN serves as a tumor suppressor and mostly found mutated in its phosphatase domain (Eng, 2003) in several cancers (Li and Sun, 1997; Steck et al., 1997) causing overactive PI3K signaling. PTEN hydrolyzes phosphatidylinositol (3,4,5)-trisphosphate (PIP3) to phosphatidylinositol (4,5)-bisphosphate (PIP2) (Figure 1). In this way, PTEN inhibits PIP3 dependent downstream signaling events like membrane recruitment and activation of AKT to prevent cell growth and proliferation. Hence, PTEN holds critical position in maintaining homeostasis through the inhibition of oncogenic transformation (Carracedo and Pandolfi, 2008).

Finally, the activated mTORC1 activates several downstream effectors mainly eukaryotic translation initiation factor 4E (eIF4E)-binding protein 1 (4E-BP1) and S6 kinase 1 (S6K1), that represses autophagy and promotes protein synthesis. S6K1 phosphorylates rictor causing mTORC2 disassembly (Dibble et al., 2009; Julien et al., 2010; Treins et al., 2010) and also degrades IRS proteins (Harrington et al., 2004; Shah et al., 2004) which dampens the PI3K mediated signaling cascade.

Mutations in the genes encoding for the proteins of mTOR associated BRN can lead to different types of cancers including sporadic cancers, hamartoma syndromes or phakomatoses, cowden syndrome (PTEN), neurofibromatosis (NF1, NF2) and peutz–Jeghers syndrome (LKB1) (Menon and Manning, 2008). Deregulation of entities in mTOR associated BRN result in certain other complications like insulin resistance and type 2 diabetes. Moreover, mTOR pathway can also be hyper-stimulated (e.g., in adipose tissues) under situation of excessive nutrients that can ultimately lead to the same complications (Um et al., 2004; Khamzina et al., 2005; Tremblay et al., 2007).

### Computational modeling

**Figure 2:**
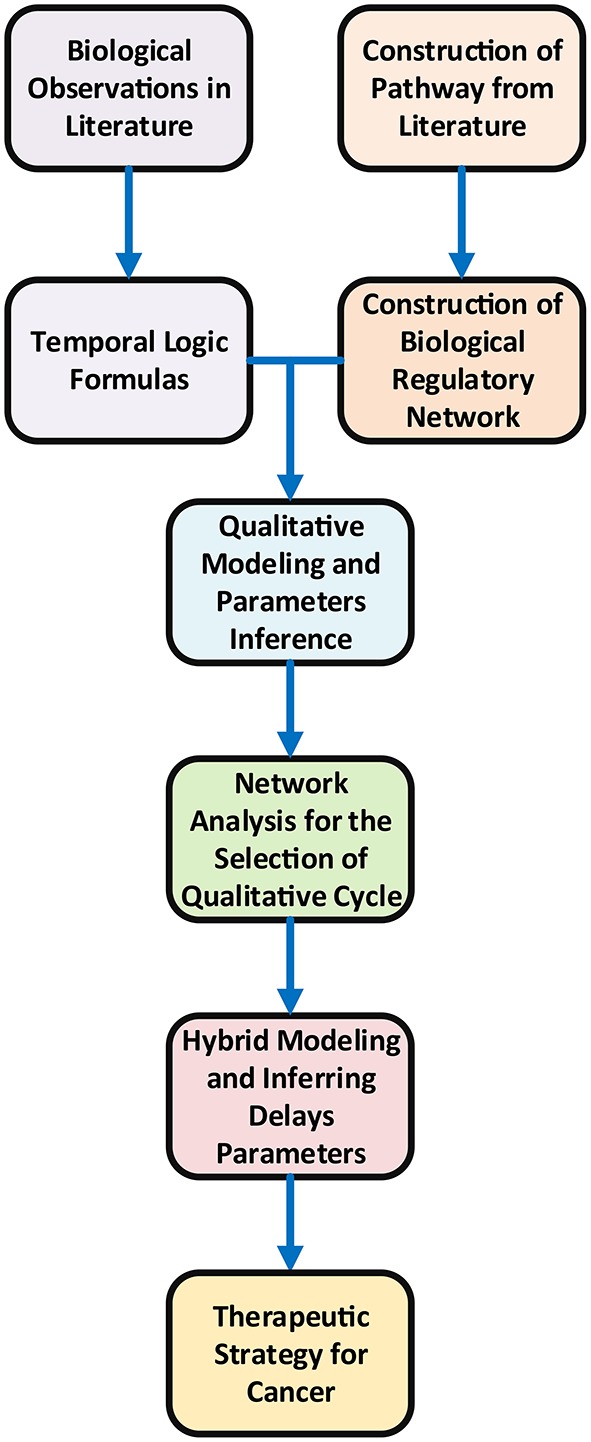
Workflow Design of the study of the therapeutic strategy for cancer.

Gene expression is a complex process and its regulation determines the overall cellular dynamics (De Jong, 2002). Computational techniques in systems biology facilitate to explore the role of genes, proteins and overall dynamics of the system (Glass and Kauffman, 1973). Qualitative modeling framework is one of the established methods to analyze gene expression dynamics (Thomas, 1978; Thomas and d'Ari, 1990; De Jong et al., 2004) in the form of biological regulatory networks (BRNs) (Lewin, 2000). A BRN is modeled by a directed graph where vertices represent biological entities e.g., DNA, RNA, proteins and other biological molecules whereas edges correspond to regulatory interactions (i.e., activation and inhibition) (Bernot et al., 2007). The design of the study is illustrated in Figure 2.

### Contributions

The main objective of this study is to build a refined computational model of mTOR regulation that could predict therapeutic targets to inhibit the progression of cancer. A BRN of mTOR and its interacting proteins (PI3K, PTEN, mTORC2, Akt, mTORC1 and FOXO) has been abstracted from the pathway (Figure 1) in order to explore the dynamics based on the logical formalism of René Thomas (Peres and Jean-Paul, 2003; Bernot et al., 2004, 2007). The unknown parameters in the logical model are inferred based on biological observations formally encoded as CTL (Computational Tree Logic) formulas in SMBioNet (Selection of Models of Biological Networks) tool (Mcadams and Shapiro, 1995). The qualitative model (State Graph) of the BRN infers the dynamics such as homeostasis in the form of qualitative cycles and stable behavior in the form of stable state (SS). The most significant qualitative cycle is selected based on the centrality values of the qualitative states in the model. A linear hybrid automaton of the selected cycle is constructed using Hytech model checker (Henzinger et al., 1997) with new parameters for production and degradation time delays. Hytech inferred the values of these parameters in the form of linear constraints. These constraints are further analyzed to infer the pairwise relations between any possible pair of delays of genes. These relations show only one significant relation between AKT and mTORC2 in terms of delays. The analysis of the hybrid model provides a formal proof that during homeostasis the inhibition time delay of Akt is less than the inhibition time delay of mTORC2. This enforces that in homeostasis Akt is degraded with a faster rate than mTORC2 which suggests that the inhibition of Akt along with the activation of mTORC2 may be a better therapeutic strategy for the treatment of cancer.

## Methods

### Reduction of signaling pathway

**Figure 3:**
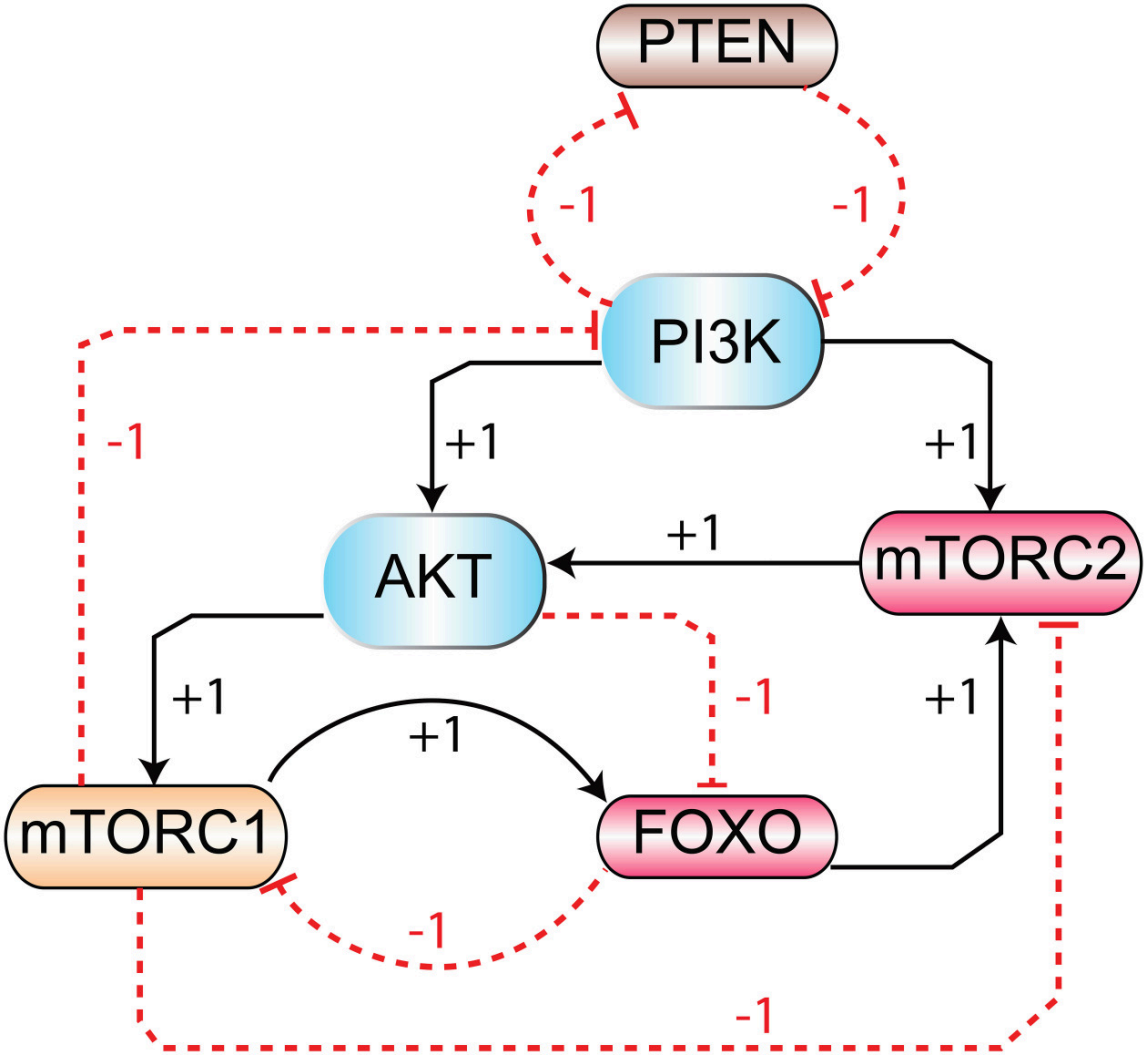
The mTOR-associated BRN: abstraction of mTOR signaling pathway (Figure 1) in BRN. Positive (activation) and negative (inhibition) regulations are represented by “+” and “−” signs respectively.

The signaling pathway shown in Figure 1 is further reduced to a BRN shown in Figure 3 by the reduction rules described in (Naldi et al., 2009; Saadatpour et al., 2013). These rules have already been applied to reduce the TLR4 and JAK/STAT signaling pathways to a BRN with all possible regulatory feedback circuits (Paracha et al., 2014). The abstracted mTOR-associated BRN is composed of six proteins which are PI3K, PTEN, mTORC2, Akt, mTORC1, and FOXO.

### Qualitative modeling

Biological regulations (production and degradation) are subjected to expression levels of entities in BRN. An entity *p*1 activates or inhibits another entity *p*2, at a specific threshold. A qualitative threshold can be described as a discrete level (first, second, third etc.). René Thomas proposed a modeling framework which assumes qualitative thresholds and parameters to derive the dynamics of a BRN. Several methods are in use to model the behavior of biological systems (Peres and Jean-Paul, 2003; Bernot et al., 2004, 2007). Continuous modeling frameworks based on ordinary and partial differential equations are widely used. These frameworks rely on precise quantitative values, which in many cases are not known. This limitation led to the development of qualitative modeling framework. Kauffman et al., introduced a logical formalism based on Boolean logic where each entity was considered as “ON” (1) or “OFF” (0) to represent its activation or inhibition, respectively (Kauffman, 1969, 1993; Somogyi et al., 1997). This approach was further extended to kinetic logic formalism by Thomas to incorporate multi-valued (0,1,2,3,…) expression levels of entities. Formal methods such as model-checking approach can help to infer the parameters of complex systems (Bernot et al., 2007). BRNs are complex systems and their parameters can be inferred with such approaches.

This study is based on the kinetic logical formalism developed by René Thomas (Thieffry and Thomas, 1995) to model the biological regulatory network (BRN) of mTOR using GENOTECH tool Ahmad (2009) (available at https://github.com/DrJamilAhmad/GENOTECH/blob/master/GenoTechE.jar). An important feature of kinetic modeling is positive or negative feedback circuits. An entity favors the activation of another entity in the BRN through positive feedback and is necessary to generate multi-stationarity (stable states), whereas an entity favoring the inhibition of another entity through negative feedback is a necessary condition to generate oscillatory behavior (homeostasis) (Thomas, 1981). Number of studies performed on genetic networks that incorporated analysis of positive and negative feedbacks with formal methods can be found in Kauffman (1993), Somogyi and Sniegoski (1996), and Szallasi and Liang (1998). Formal definitions provided in Aslam et al. (2014) and Paracha et al. (2014) can be obtained for detailed description.

### Parameters inference using model checking

Qualitative dynamics of Thomas networks depend on the values of logical parameters which are unknown a priori. These parameters are used to render system dynamics as a directed state graph (discrete or qualitative model), which incorporates important behaviors such as cycles or stable states. The inference of biologically coherent parameters is an important aspect of qualitative modeling. In this direction, Bernot et al., introduced an approach to infer these logical parameters using a formal method approach called *model checking*. This approach is implemented in SMBioNet (Selection of Models of Biological Networks) tool. It performs an exhaustive enumeration of models and selects those set of parameters which are consistent with experimental observations expressed as temporal logic formulas. Similar parameter estimation approach (by using SMBioNet tool) has been employed to study qualitative behavior of several biological systems including immunity control mechanism in lambda phage network (Mcadams and Shapiro, 1995), pathogenesis and clearance mechanism of dengue virus (Aslam et al., 2014), MAL-Associated network of Cerebral Malaria (Ahmad et al., 2012) and the role of OGT in Cancer progression (Saeed et al., 2016).

### Network analysis

Graph-theoretic approaches have been successfully applied on large protein networks (Barabasi and Oltvai, 2004; Stelniec-Klotz et al., 2012). The state graph can be further analyzed using network analysis techniques in terms of graph connectivity (Junker and Schreiber, 2011) by sorting it on the basis of maximum betweenness centrality (Freeman, 1977). The states with higher betweenness centralities represent higher chances of their occurrences. This may in terms of biological phenomenon represent the entities with frequent expressions. The qualitative states in the model with high betweenness centralities are compared to the rest of the state space in order to identify most favorable cycle (Tareen et al., 2015; Saeed et al., 2016).

### Hybrid modeling

**Figure 4:**
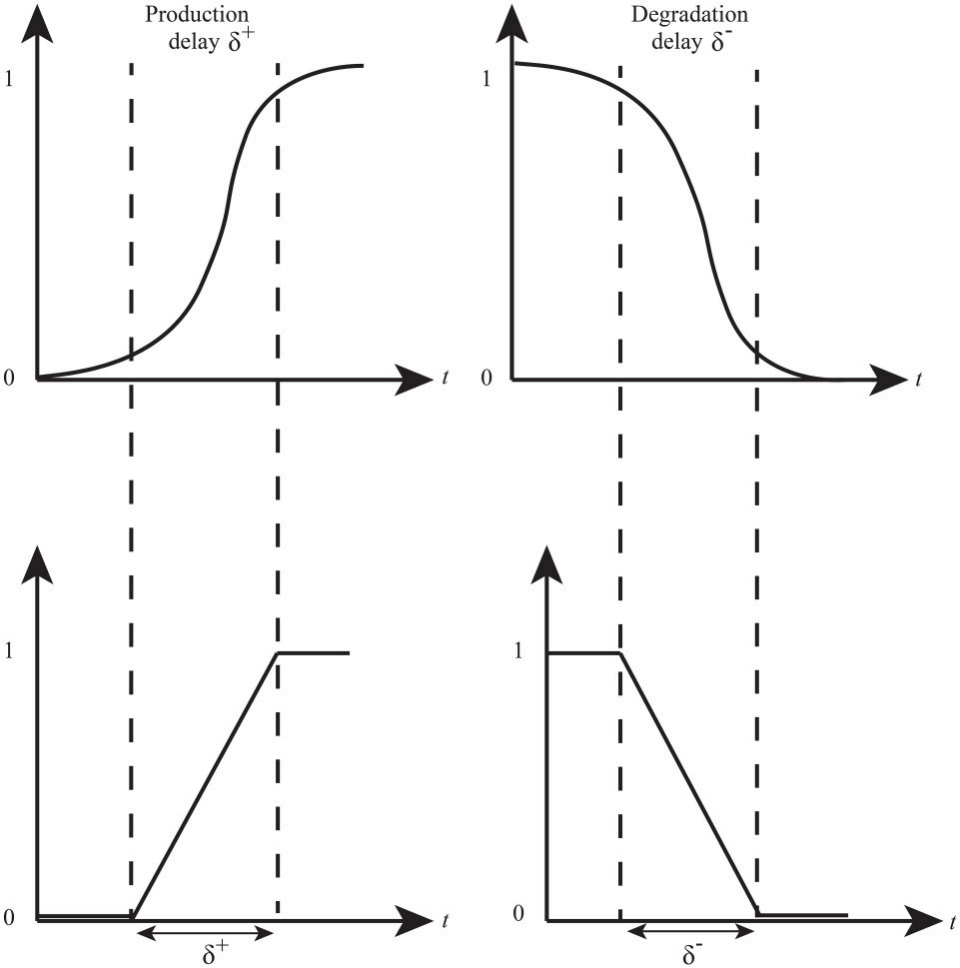
Time delays in expression evolution Ahmad et al. (2012). The evolution of qualitative states is characterized by time delays: δ^+^ represents production delay or time required to pass from low level to next high level whereas δ^−^ is the time delay required for a gene to pass from high level to low level (degradation delay).

**Figure 5:**
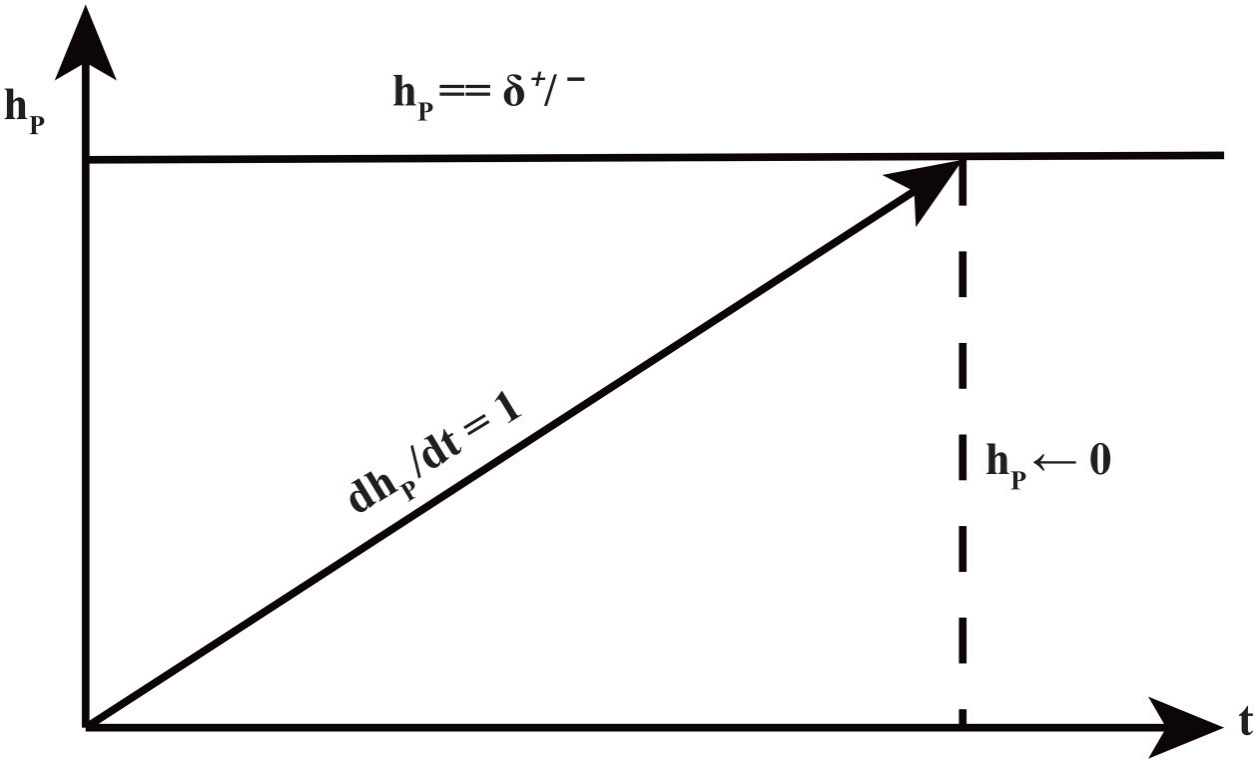
The production and degradation time delays δ^+/−^ associated with an entity *p*. The clock *h*_*p*_ measures the production or degradation time delays.

René Thomas' framework provides useful insights into the discrete qualitative behavior of a biological system. However, naturally, the expression levels of proteins evolve in a continuous manner. Hybrid modeling combines discrete changes of a system with continuous changes (differential equation) in a single formalism Bio-Linear Hybrid Automaton (Bio-LHA) has been proposed for the hybrid modeling of qualitative BRNs (Ahmad et al., 2007). Bio-LHA uses time delays along with continuous variables (clocks) to compute production and degradation time of gene expressions. Production (δ^+^) or degradation (δ^−^) delay is the time required for a gene expression to reach from a lower level to a higher level or vice versa (Figure 4). In this approach, a clock variable (*h*) is associated with each entity which is initially set to zero and it evolves with rate 1 when the expression evolves. A clock is reset when it measures a production or degradation delay as shown in Figure 5. Hybrid model checking tool such as HyTech (Henzinger et al., 1997) can be used to infer the values of delays in the form of linear delay constraints for behaviors (paths toward stable states and cycles) observed in the qualitative model. Invariance kernel (Ahmad et al., 2007; Ahmad and Roux, 2010) represents cycles in the hybrid models which can also be characterized with delay constraints. These delay constraints are further converted into the relation matrix in order to find constraints between any two types of delays (production or degradation) of all entities. This modeling approach has been successfully applied to model a variety of BRNs (Ahmad et al., 2007, 2008, 2012; Ahmad, 2009; Aslam et al., 2014; Bibi et al., 2016).

## Results

### Parameters inference

To construct qualitative model of the mTOR associated BRN, SMBioNet tool has been employed to correctly estimate logical parameters according to biologicalobservations in literature (Chen et al., 2010; Zoncu et al., 2011; Laplante and Sabatini, 2012). This tool takes Computation Tree Logic (CTL) formulas representing biological observations and BRN as inputs and selects those sets of parameters which verify these formulas. In BRN modeling these parameters are used to incorporate behaviors in the form of paths, cycles and stable states (SS) as specified in the CTL formulas (Table 1). Formula 1 in Table 1 represents that in a particular homeostatic behavior (represented by CTL operator *E*) all entities with 0 expression levels after next qualitative state (represented by CTL operator *X*) finally reach the same expression levels in future (represented by CTL operator *F*). Formula 2 represents the biological observation that the overexpression of PTEN, FOXO, PI3K, mTORC1, and inactivation of Akt gradually leads to (represented by CTL operator ⇒) a steady carcinogenic state (represented by CTL operators *F* and *G*) with the overexpression of Akt, mTORC1, mTORC2, PI3K, and inactivation of PTEN and FOXO. The effect of PTEN inhibition on Akt/mTORC1 pathway eventually leads to a SS where FOXO and PTEN are not expressed. This behavior is encoded by Formula 3. On the basis of CTL formulas, SMBiogenerated eight sets of logical parameters for mTOR associated BRN (see Supplementary Files 1, 2).

**Table 1:** CTL formulas.

**No**.	**CTL Formulas**	**References**
1	(*PI*3*K* = 0∧*FOXO* = 0∧*mTORC*2 = 0, ∧*Akt* = 0∧*mTORC*1 = 0∧*PTEN* = 0) →, (*EX*(*EF*(*PI*3*K* = 0∧*FOXO* = 0∧*mTORC*2 = 0∧*Akt* = 0∧*mTORC*1 = 0)))	Zoncu et al., 2011
2	(*PTEN* = 1∧*FOXO* = 1∧*Akt* = 0∧*PI*3*K* = 1∧*mTORC*1 = 1∧*mTORC*2 = 1) → (*EF*(*AG*(*Akt* = 1∧*PTEN* = 0∧*FOXO* = 0∧*mTORC*1 = 1∧*mTORC*2 = 1∧*PI*3*K* = 1)))	Carracedo and Pandolfi, 2008; Chen et al., 2010; Zoncu et al., 2011; Laplante and Sabatini, 2012
3	(*PI*3*K* = 1∧*FOXO* = 0∧*Akt* = 1∧*mTORC*1 = 1) → (*EF*(*AG*(*Akt* = 1∧*FOXO* = 0∧*PTEN* = 0)))	Carracedo and Pandolfi, 2008; Zoncu et al., 2011

*These CTL formulas are used in SMBioNet tool to infer the set of logical parameters. Formula 1 is designed to observe homeostasis while the other two formulas are used for observing stable steady state(s)*.

### Selection of a qualitative model

The eight qualitative models of these sets were further analyzed for cycle(s) and SS(s) using GENOTECH tool. Almost all the parameter sets provide comparable results, revealing similar cycles and SS(s). First four models were selected on the basis of biological plausible SS (1, 0, 1, 1, 1, 0) representing the activation and inactivation states of entities in order of PI3K, PTEN, mTORC2, Akt, mTORC1 and FOXO, respectively. This SS represents the activation of PI3K, mTORC2, Akt and mTORC1 along with the inactivation of PTEN and FOXO. Subsequently, these 4 models were further compared for the logical parameter values which are coherent with biological observations. The set of logical parameters for this model (Supplementary File 3) is given in the last column of Table 2. The selected model (Figure 3) also shows eight cycles along with one SS (1, 0, 1, 1, 1, 0).

**Table 2:** Selection of logical parameters.

**Parameter**	**Resource(s)**	**Range of Values**	**Selected Parameters**
*K*_*PI*3*K*_	{}	0	0
*K*_*PI*3*K*_	{*mTORC*1}	0,1	1
*K*_*PI*3*K*_	{*PTEN*}	0,1	1
*K*_*PI*3*K*_	{*mTORC*1, *PTEN*}	0,1	1
*K*_*PTEN*_	{}	0	0
*K*_*PTEN*_	{*PI*3*K*}	1	1
*K*_*mTORC*2_	{}	0	0
*K*_*mTORC*2_	{*FOXO*}	0,1	1
*K*_*mTORC*2_	{*mTORC*1}	1	1
*K*_*mTORC*2_	{*PI*3*K*}	0,1	1
*K*_*mTORC*2_	{*FOXO, mTORC*1}	1	1
*K*_*mTORC*2_	{*FOXO, PI*3*K*}	0,1	1
*K*_*mTORC*2_	{*mTORC*1, *PI*3*K*}	1	1
*K*_*mTORC*2_	{*FOXO, mTORC*1, *PI*3*K*}	1	1
*K*_*Akt*_	{}	0	0
*K*_*Akt*_	{*mTORC*2}	0	0
*K*_*Akt*_	{*PI*3*K*}	0	0
*K*_*Akt*_	{*mTORC*2, *PI*3*K*}	1	1
*K*_*mTORC*1_	{}	0	0
*K*_*mTORC*1_	{*Akt*}	0,1	0
*K*_*mTORC*1_	{*FOXO*}	1	1
*K*_*mTORC*1_	{*Akt, FOXO*}	1	1
*K*_*FOXO*_	{}	0	0
*K*_*FOXO*_	{*Akt*}	1	1
*K*_*FOXO*_	{*mTORC*1}	0	0
*K*_*FOXO*_	{*Akt, mTORC*1}	1	1

*This table describes the ranges of logical parameter values. Some parameters are fixed to single values 0 or 1 based on biological observations. The last column lists one of the eight selected parameter sets generated by SMBioNet*.

**Figure 6:**
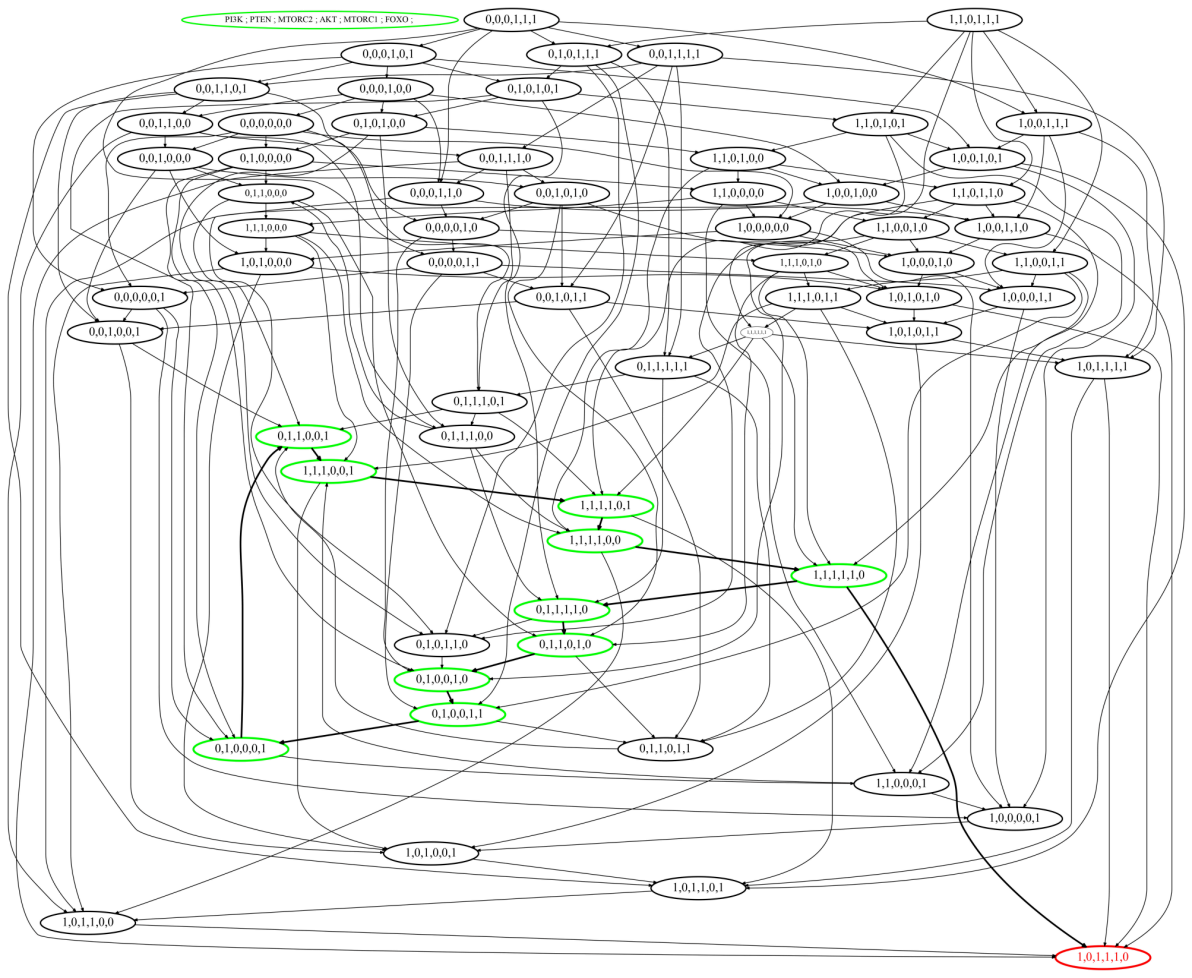
State graph of the mTOR-associated BRN. Nodes of the state graph represent the states of the BRN and edges between states represent the evolution of states. A state in this graph shows the expressions of the entities PI3K, PTEN, mTORC2, Akt, mTORC1, and FOXO respectively. The stable steady state (1,0,1,1,1,0) represents high expression levels of all entities except tumor suppressors. Cycle having maximum betweenness centrality is represented in green color with bold arrows, also showing bifurcation toward stable state (1,0,1,1,1,0).

Several states lead the BRN directly into SS which represent critical divergence toward disease. All such states that eventually progress toward deadlock state do not possess functional PTEN or FOXO while having cellular proliferatory elements fully activated, e.g., (1,1,1,1,1,0), (1,0,1,1,1,1) as represented in (Figure 6). Thus, the down regulation of these tumor suppressor genes (PTEN and FOXO) bring this deadlock (SS) where no regulator is present to perform its function. The states (1,0,1,1,0,0), (0,0,1,1,1,0), (1,0,0,1,1,0), and (1,0,1,0,1,0) with temporary inhibition of PI3K, mTORC2, Akt, or mTORC1 are restrained in proceeding states resulting in their full and uncontrollable activation. This impact of tumor suppressor gene can be observed in cyclic state (1,1,1,1,1,0) that progresses into (0,1,1,1,1,0) (Figure 6) where PI3K is down-regulated through the inhibitory effect of PTEN to slow down further increase in cell mass and number. So the pre-occupation of PTEN is desired to recover the system into homeostatic state (0,1,1,1,1,0) that otherwise could divert into SS (1,0,1,1,1,0). The selected cyclic trajectory along with its respective constraints is given in **Figure 8**, specifies the stay conditions for each cyclic state and its violation would activate counter mechanism of autophagic inhibition by mTORC1 leading to cancer.

### Validation of qualitative model with ASP

We applied exhaustive model-checking to validate the qualitative model of this study as proposed in Ben Abdallah et al. (2015). In this approach, the authors present a logical approach (using the Answer Set Programming language (ASP) Baral, 2003) to simulate and exhaustively analyze the dynamics of multivalued biological regulatory networks. The ASP method searches the attractor basins (stable states) which the region from which it is not possible to exit. By translating the model of Figure 3 to an automata network (Supplementary Files 6–9) and giving it as an input to the method of Ben Abdallah et al. (2015), we found that the set of all the attractor basins is reduced to a single stable state: (*PI*3*K* = 1, *AKT* = 1, *PTEN* = 0, *MTORC*2 = 1, *FOXO* = 0, *MTORC*1 = 1). This result is effectively coherent with the qualitative model given in Figure 6.

### Selection of cycle

**Figure 7:**
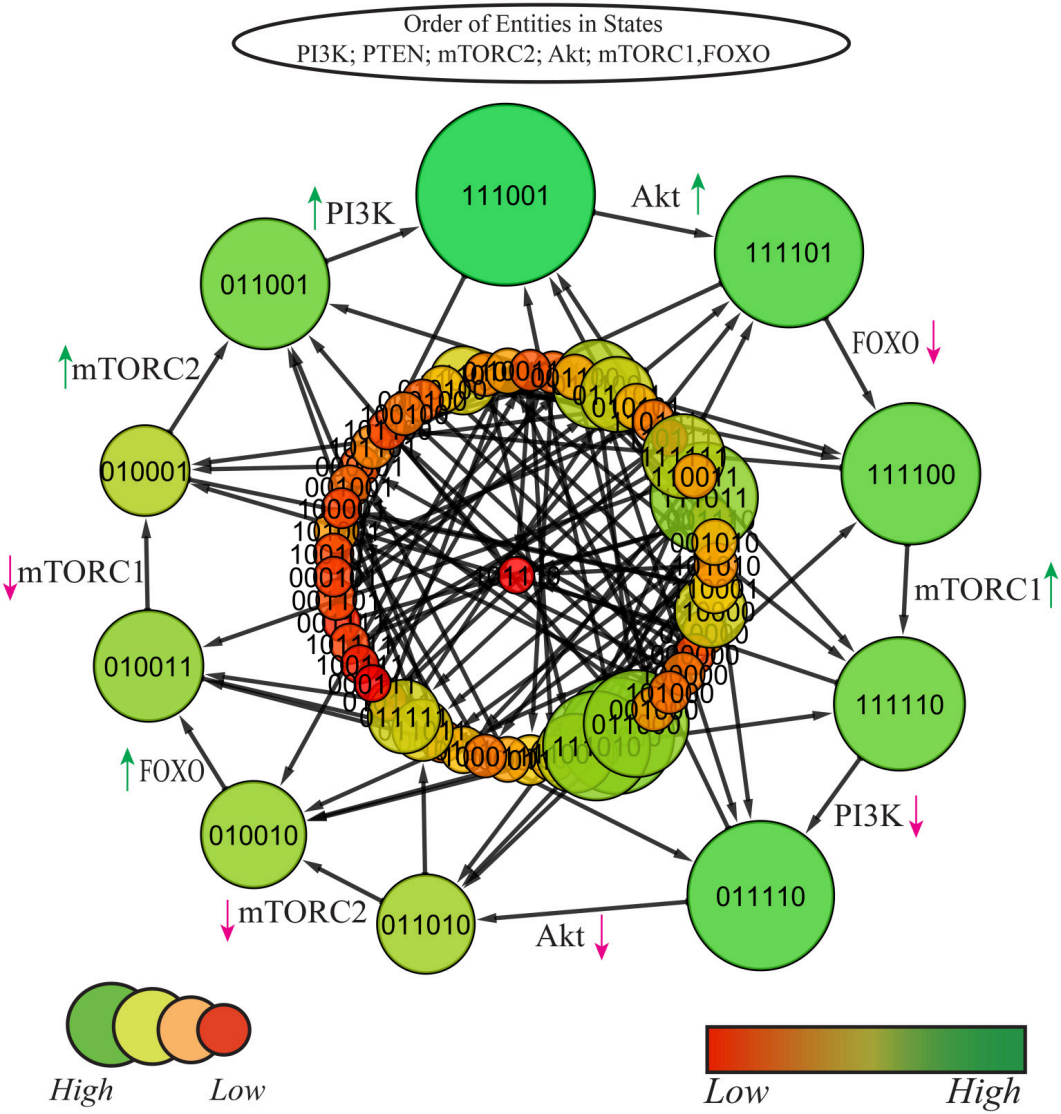
State graph of mTOR associated BRN: size and color of nodes are scaled on the basis of betweenness centrality, larger nodes represent higher centralities with variable shades of green color (darker the color, higher is the centrality). The graph comprises of 64 states and 192 edges. Cycle with maximum betweenness centrality is extracted out at top layer. Red and green arrows represent degradation and production of entities, respectively.

Since the model shows eight cycles therefore it is important to identify the most probable biological cycle. Thus, on the basis of betweenness centrality a cycle was computed by using Cytoscape tool (Shannon et al., 2003) that sorts all the states on the basis of their betweenness centralities (Freeman, 1977; Tareen et al., 2015), as presented in Figure 7 (Supplementary File 4). The nodes with larger diameter represent states with higher betweenness centrality. The cycle with maximum betweenness centrality: (1, 1, 1, 0, 0, 1) → (1, 1, 1, 1, 0, 1) → (1, 1, 1, 1, 0, 0) → (1, 1, 1, 1, 1, 0) → (0, 1, 1, 1, 1, 0) → (0, 1, 1, 0, 1, 0) → (0, 1, 0, 0, 1, 0) → (0, 1, 0, 0, 1, 1) → (0, 1, 0, 0, 0, 1) → (0, 1, 1, 0, 0, 1) → (1, 1, 1, 0, 0, 1)shows oscilation of all entities except PTEN. The cycle reveals that the constant activation of PTEN is required for homoeostasis. Any diversion from this cycle would either lead toward carcinogenic SS (1,0,1,1,1,0). All the cyclic trajectories represented in Figure 6 show expression of PTEN that positively regulates PI3K and enforces the model to remain in homeostasis. On the contrary, uncontrolled expression of PI3K and subsequent stimulation of cellular proliferative machinery mainly Akt and mTORC1 would either cause diabetic disorders or more severe circumstances like oncogenesis.

### Hybrid model

**Figure 8:**
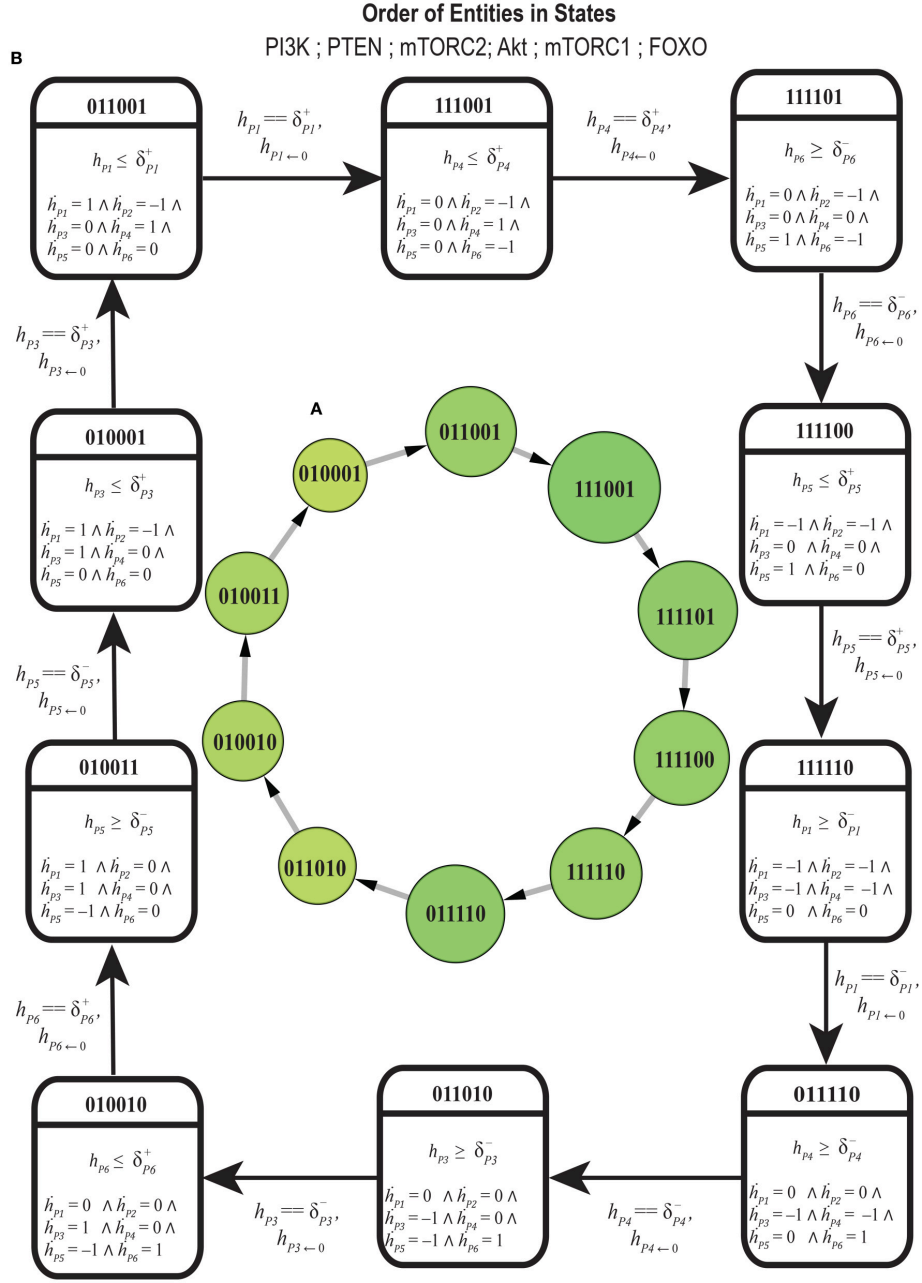
**(A)** Discrete representation of cycle selected on the basis of centrality, larger circles represent higher centrality states. **(B)** Bio Linear Hybrid Automata of selected cycle: Each square symbol represents a hybrid location by capturing the discrete expression dynamics (top) and the continuous evolution of all clocks. Clock value *h* resets to 0 after each transition. P1 = PI3K, P2 = PTEN, P3 = mTORC2, P4 = Akt, P5 = mTORC1, P6 = FOXO.

The cycle in Figure 7 shows homeostatic biological regulation of entities in the form of the switching of their low and high expression levels. In the cycle, the stable high expression of PTEN gene is revealed as a mandatory condition in all the states to maintain homeostasis (represented by 1 expression level in all cyclic states) while the expression of other entities oscillate in relation to each other. The Bio-LHA model in Figure 8 of this cycle was implemented in HyTech tool (Supplementary File 5) in order to predict its underlying causality relations of delays by analyzing the invariance kernel (Ahmad et al., 2008). The invariance kernel represents a set of viable cyclic trajectories in the state space of the Bio-LHA. Delay constraints characterizing the invariance kernel of the selected cycle were computed by HyTech tool (Table 3). In Table 3, the notation π is used to represent the sum of production and degradation delays as period (Ahmad, 2009). Conjunctions of all these constraints (1–9) constitute a necessary and sufficient condition for the existence of the invariance kernel and hence the qualitative cycle. Violation of any constraint would result in a null invariance kernel and eventually the qualitative cycle will no more exist. It is therefore sufficient that all the constraints should be valid (true) for the existence of the invariance kernel or qualitative cycle. For example, in Table 3, constraint 1 ($\delta_{FOXO}^{+}\leq |\delta_{mTORC1}^{-}|+|\delta_{Akt}^{-}|$) shows that the production (activation) of FOXO is required before the degradation of Akt and mTORC1 and hence constitute a necessary condition for cycle (homeostasis). Again constraint 2 establishes another necessary condition for the existence of the cycle that explains that the degradation of FOXO should occur earlier than the production of mTORC1 and Akt. Similarly, other remaining constraints imposes necessary conditions for the existence of the cycle.

**Table 3:** Delay constraints.

**Number**	**Delay Constraints**
1	$\delta_{FOXO}^{+}\leq |\delta_{mTORC1}^{-}|+|\delta_{Akt}^{-}|$
2	$|\delta_{FOXO}^{-}|\leq \delta_{Akt}^{+}+\delta_{mTORC1}^{+}$
3	$\left|\delta_{PI3K}^{-}|\leq \delta_{mTORC1}^{+}+|\delta_{Akt}^{-}\right|$
4	$\pi \left(FOXO\right)+|\delta_{PI3K}^{-}|\leq ,\pi \left(Akt\right)+\pi \left(mTORC1\right)$
5	$\pi \left(FOXO\right)+\pi \left(PI3K\right)\leq \pi \left(Akt\right)+\pi \left(mTORC1\right)+\delta_{mTORC2}^{+}$
6	$\delta_{FOXO}^{+}+|\delta_{PI3K}^{-}|\leq \pi \left(mTORC1\right)+|\delta_{Akt}^{-}|$
7	$\pi \left(FOXO\right)+\pi \left(PI3K\right)\leq \pi \left(mTORC2\right)+\delta_{Akt}^{+}+\delta_{mTORC1}^{+}$
8	$\delta_{PI3K}^{+}+\delta_{FOXO}^{+}\leq \delta_{Akt}^{+}+\pi \left(mTORC2\right)$
9	$\pi \left(PI3K\right)+\delta_{FOXO}^{+}\leq \delta_{Akt}^{+}+\pi \left(mTORC2\right)+\delta_{mTORC1}^{+}$

*Delay Constraints of the selected cycle characterizing its invariance kernel. δ^+^ represents production (activation) delay while δ^−^ represents degradation (inhibition) delay. Notation π(e) refers to the sum of production and degradation delays of the entity e*.

From the delay constraints in Table 3, a relation matrix is derived in Table 4 containing pairwise relations of delays of the entities FOXO, PI3K, mTORC1, mTORC2, and Akt. A relation between a pair of delays with both ≤ and ≥ reveals that the cycle is desensitized to the violation of such constraints. The table contains only one pairwise constraint between the degradation delays of Akt and mTORC2. It is important to note that these relationships are enforcing homeostasis represented by the qualitative cycle. From the results, it can be implicated that if cellular systems tends to escape homeostasis it may lead toward pathogenesis.

**Table 4:** Relation matrix.

	**$\delta_{FOXO}^{+}$**	**$\left|\delta_{FOXO}^{-}\right|$**	**$\delta_{PI3K}^{+}$**	**$\left|\delta_{PI3K}^{-}\right|$**	**$\delta_{mTORC1}^{+}$**	**$\delta_{mTORC1}^{-}$**	**$\delta_{mTORC2}^{+}$**	**$\left|\delta_{mTORC2}^{-}\right|$**	**$\delta_{Akt}^{+}$**	**$\left|\delta_{Akt}^{-}\right|$**
$\delta_{FOXO}^{+}$	=									
$\left|\delta_{FOXO}^{-}\right|$	≤, ≥	=								
$\left|\delta_{PI3K}^{-}\right|$	≤, ≥	≤, ≥	=							
$\delta_{PI3K}^{+}$	≤, ≥	≤, ≥	≤, ≥	=						
$\delta_{mTORC1}^{+}$	≤, ≥	≤, ≥	≤, ≥	≤, ≥	≤, ≥	=				
$\left|\delta_{mTORC1}^{-}\right|$	≤, ≥	≤, ≥	≤, ≥	≤, ≥	≤, ≥	=				
$\delta_{mTORC2}^{+}$	≤, ≥	≤, ≥	≤, ≥	≤, ≥	≤, ≥	≤, ≥	=			
$\left|\delta_{mTORC2}^{-}\right|$	≤, ≥	≤, ≥	≤, ≥	≤, ≥	≤, ≥	≤, ≥	≤, ≥	=		
$\delta_{Akt}^{+}$	≤, ≥	≤, ≥	≤, ≥	≤, ≥	≤, ≥	≤, ≥	≤, ≥	≤, ≥	=	
$\left|\delta_{Akt}^{-}\right|$	≤, ≥	≤, ≥	≤, ≥	≤, ≥	≤, ≥	≤, ≥	≤, ≥	≤	≤, ≥	=

*Relation matrix of the selected cycle that shows the pairwise relationships of delays of the entities FOXO, PI3K, mTORC1, mTORC2 and Akt*.

The only pairwise relation between $\delta_{Akt}^{-}$ and $\delta_{mTORC2}^{-}$ (Table 4) reveals a significant property of the selected homeostatic cycle where the degradation delay of Akt is less than or equal to the degradation delay of mTORC2. In other words, the degradatin of AKT occurs at faster rate than the degrdation of mTORC2. Interestingly, this is the only observed property of the cycle that enforces its existence dramatically. Thus, this constraint provides a governing rule that if violated may bifurcate the trajectory toward stable steady state (1,0,1,1,1,0) (represented by red colored state in Figure 6).

## Discussion

The pathological roles of PTEN, mTOR, and Akt have been well established in different diseases including diabetes and different types of cancer (Altomare and Testa, 2005; Zoncu et al., 2011; Hopkins et al., 2014). The risk for the development of cancer in diabetic patients is increased with hyperinsulinemia and oxidative stress (Vigneri et al., 2009). With nutrient uptake, levels of growth factors and hormones rise in the blood stream that triggers certain biochemical processes. Feeding promotes insulin levels in the bloodstream that binds to its particular receptors causing stimulation of PI3K downstream signaling. Deregulation of PI3k/Akt mediated mTOR signaling pathway contributes to insulin resistance and associated conditions (Harrington et al., 2004; Shah et al., 2004). PI3K tends to stimulate mTORC2 and both of these proteins initiate activation of Akt (Alessi et al., 1997; Sarbassov et al., 2005). Subsequently, Akt favors mTORC1 activation by phosphorylating TSC1/TSC2 complex (Zoncu et al., 2011). mTORC1 is able to impair insulin signaling via its substrates S6K1 which then phosphorylates serine residues of IRS1 causing downregulation of PI3K/Akt pathway (Harrington et al., 2004; Shah et al., 2004). In this way, mTORC1 activity can contribute to insulin resistance. Therefore, it is important to identify therapeutically favorable regulatory event in mTOR-associated BRN that plays a major role in triggering pathological signaling cascade (Vigneri et al., 2009).

Formal methods are widely applicable for the correctness of ICT Systems due to their computational ability of rigorous testing. For the last few decades, formal methods have been successfully used for the modeling and verification of complex biological systems (Kitano, 2002). Kinetic Logic formalism is a well-known approach for the qualitative modeling of a BRN that deciphers its qualitative dynamics in the form of a directed graph, where a node represents a qualitative state and an edge represents an evolution from one state to its successor state (Thomas, 1979; Thomas and d'Ari, 1990; Thomas et al., 1995). Since the qualitative model ignores the time in the evolution of expression levels, a hybrid model is built in order to ensure that evolutions due to activation or inhibition are taking place after production and degradation delays (Ahmad et al., 2007, 2008; Ahmad, 2009). Of course, these delays are un-known and are treated as unvalued parameters in the hybrid model. Consequently, any behavior captured in the qualitative model (cycle or path) can be temporally verified against the production and degradation time delay parameters by using the hybrid model checker HyTech (Henzinger et al., 1997) that automatically synthesizes the values of parameters (delays) in the form linear parametric constraints. Moreover, this approach has been successfully applied on a variety of BRNs for the temporal analysis of their behaviors (Ahmad et al., 2012; Aslam et al., 2014; Saeed et al., 2016).

The qualitative model (state graph) of the mTOR-associated BRN predicted cycles and a stable state. The most biologically probable cycle was selected that shows the oscillation of PI3K, mTORC2, Akt, mTORC1, and FOXO while PTEN is constantly expressed (level 1). On the other side, simultaneous deactivation of PTEN and FOXO along with the activation of Akt, PI3K, mTORC1 and mTORC2 tends to maintain the system in a stable state (1, 0, 1, 1, 1, 0). The same pattern of activation and deactivation of entities has also been observed in diabetes and different types of cancers (Altomare and Testa, 2005; Hopkins et al., 2014).

Genes' expression goes through various levels (low and high) under regulatory mechanism to maintain homeostasis. The regulation of the expressions of PI3K is under the regulatory mechanism of PTEN and mTORC1 that has been found perturbed in almost all cancer types (Hopkins et al., 2014). Downregulated PTEN has deleterious impacts on cell cycle regulation, growth and survival. In the stable state of the qualitative model PTEN is downregulated while PI3K is found overexpressed. In recent studies, PTEN has been demonstrated to downregulate the activity of mTORC1 through various pathways (Sonntag et al., 2012) which is also evident in the qualitative cycle. However, in the stable state of the qualitative model, PTEN is constantly downregulated and mTORC1 is thus overexpressed. These findings opens up various aspects of future exploration for the role of PTEN in hyperinsulinimia.

The hybrid model of the selected cycle predicted the time delays of the entities to maintain homeostasis. The pairwise relationships of delays suggest one unique pattern of faster Akt degradation than mTORC2 degradation for maintaining homoeostasis. It also suggests that therapeutics must be designed based on the fact that Akt must be cleared out of the system as soon as it performs its function along with keeping a slower degradation rate for mTORC2. This also eliminates the risk of prolong Akt activation that may hyper-activate downstream signaling cascade. Another important fact that is perceived through this constraint relationship is that mTORC1 has to be suppressed (under cancerous circumstances) to reduce its inhibitory effect upon mTORC2 which would prevent early degradation of mTORC2 as compared to Akt. This trend would keep an equilibrium between cellular proliferative elements PI3K, Akt, mTORC1, and that of apoptotic factors (e.g., FOXO). Based on these observations, further wet-lab exploration for the roles of PTEN, mTORC1, mTORC2, and Akt is required in the perspective of targeting cancer cell proliferation.

## Conclusion

In last few decades, understanding of the glucose metabolism in both proliferating cancer and normal cells is studied extensively. PI3K, Akt and mTOR play significant roles in metabolism and their deregulation can lead to different cancers. In this context, the regulatory network of these entities has been modeled and analyzed to explore its dynamics. Discrete and hybrid models have been constructed to predict the qualitative and timed dependent behaviors. In the qualitative model, cycles representing homeostasis and a stable state representing the disease state have been predicted. The most biologically probable cycle represents that the expression levels of the entities except PTEN should oscillate to maintain homeostasis. Moreover, the cycle states show the constant expression (level 1) of PTEN. On the other hand in the stable state, PI3K, mTORC2, Akt, and mTORC1 are always overexpressed while PTEN and FOXO are constantly down regulated which can ultimately lead to cancer. The hybrid model revealed the time delay constraints of the most biologically probable cycle. Further analysis of the constraints predicted the pairwise relations between the production and degradation time delays of all the entities. One relation highlighted that during homeostasis, the inhibition time delay of Akt is less than the inhibition time delay of mTORC2. In conclusion, our observations characterize that during homeostasis, Akt is degraded with a faster rate than mTORC2 which further suggests that this inhibition of Akt along with the activation of mTORC2 may be exploited for a better therapeutic strategy against cancer.

## Authors contributions

ZB and JA conceived and designed the experiments, performed the experiments, analyzed the data, contributed reagents/materials/analysis tools, wrote the paper, prepared figures and/or tables, reviewed drafts of the paper. AS and RP analyzed the data, prepared figures and/or tables, wrote the paper, reviewed drafts of the paper. TS, AA, HJ, and SU analyzed the data, reviewed drafts of the paper, technical Support. EB, and OR analyzed the data, contributed reagents/materials/analysis tools, wrote the paper, reviewed drafts of the paper.

### Conflict of interest statement

The authors declare that the research was conducted in the absence of any commercial or financial relationships that could be construed as a potential conflict of interest.

## Supplementary material

Supplementary File 1 Qualitative models with logical parameters (SMBioNet code).

Supplementary File 2 SMBioNet output file.

Supplementary File 3 mTOR BRN (implemented in GENOTECH).

Supplementary File 4 Cytoscape file (State graph sorted on the basis of betweenness centrality).

Supplementary File 5 HyTech input file.

Supplementary File 6 mTOR model represented with Automata Network (AN) formalism.

Supplementary File 7 mTOR Automata network figure.

Supplementary File 8 Automata Network (AN) file of mTOR BRN in Answer Set Programming (ASP).

Supplementary File 9 Answer Set Programming (ASP) results.
