# Supplementary material for: Formal Modeling of mTOR Associated Biological Regulatory Network Reveals Novel Therapeutic Strategy for the Treatment of Cancer
Source: Front Physiol. 2017 Jun 13;8:416. doi: 10.3389/fphys.2017.00416 (PMC5468443; doi:10.3389/fphys.2017.00416)
Supplement: Supplementary File 1 — Qualitative models with logical parameters (SMBioNet code). [file DataSheet1.ZIP › Supplementary file 1.docx]

VAR

PI3K = 0 1 ;
MTORC2 = 0 1 ;
AKT = 0 1 ;
MTORC1 = 0 1 ;
FOXO = 0 1 ;
PTEN = 0 1 ;

REG

PTEN [(PTEN<1)]=> PI3K ;
MTORC1 [(MTORC1<1)]=> PI3K ;
PI3K [(PI3K>=1)]=> MTORC2 AKT ;
MTORC1 [(MTORC1<1)]=> MTORC2 ;
FOXO [(FOXO>=1)]=> MTORC2 ;
MTORC2 [(MTORC2>=1)]=> AKT ;
AKT [(AKT>=1)]=> MTORC1 ;
FOXO [(FOXO<1)]=> MTORC1 ;
AKT [(AKT<1)]=> FOXO ;
MTORC1 [(MTORC1>=1)]=> FOXO ;
PI3K [(PI3K<1)]=> PTEN ;

PARA

# Parameters for PI3K

K_PI3K = 0 ;
K_PI3K+MTORC1 = 0 1 ;
K_PI3K+PTEN = 0 1 ;
K_PI3K+MTORC1+PTEN = 1 ;

# Parameters for MTORC2

K_MTORC2 = 0 ;
K_MTORC2+FOXO = 0 1 ;
K_MTORC2+MTORC1 = 1 ;
K_MTORC2+PI3K = 0 1 ;
K_MTORC2+FOXO+MTORC1 = 1 ;
K_MTORC2+MTORC1+PI3K = 1 ;
K_MTORC2+FOXO+PI3K = 0 1 ;
K_MTORC2+FOXO+MTORC1+PI3K = 1 ;

# Parameters for AKT

K_AKT = 0 ;
K_AKT+MTORC2 = 0 ;
K_AKT+PI3K = 0 ;
K_AKT+MTORC2+PI3K = 1 ;

# Parameters for MTORC1

K_MTORC1 = 0 ;
K_MTORC1+AKT = 0 1 ;
K_MTORC1+FOXO = 1 ;
K_MTORC1+AKT+FOXO = 1 ;

# Parameters for FOXO

K_FOXO = 0 ;
K_FOXO+AKT = 1 ;
K_FOXO+MTORC1 = 0 ;
K_FOXO+AKT+MTORC1 = 1 ;

# Parameters for PTEN

K_PTEN = 0 ;
K_PTEN+PI3K = 1 ;

CTL

(((PI3K=0&FOXO=0&MTORC2=0&AKT=0&MTORC1=0&PTEN=0)->(EX(EF(PI3K=0&FOXO=0&MTORC2=0&AKT=0&MTORC1=0))))
&
((PTEN=1&FOXO=1&AKT=0&PI3K=1&MTORC1=1&MTORC2=1)->(EF(AG(AKT=1&PTEN=0&FOXO=0&MTORC1=1&MTORC2=1&PI3K=1))))
&
((PI3K=1&FOXO=0&AKT=1&MTORC1=1)->(EF(AG(AKT=0&MTORC1=0&FOXO=1)))->(EF(AG(AKT=1&FOXO=0&PTEN=0)))))
