# Supplementary material for: Formal Modeling of mTOR Associated Biological Regulatory Network Reveals Novel Therapeutic Strategy for the Treatment of Cancer
Source: Front Physiol. 2017 Jun 13;8:416. doi: 10.3389/fphys.2017.00416 (PMC5468443; doi:10.3389/fphys.2017.00416)
Supplement: Supplementary File 1 — Qualitative models with logical parameters (SMBioNet code). [file DataSheet1.ZIP › Supplementary file 7.pdf]

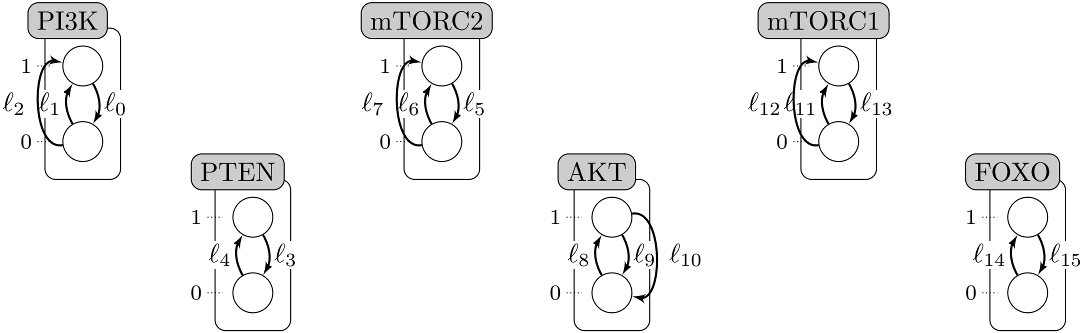

$\ell_0 = \{PTEN_1, mTORC1_1\}$
$\ell_1 = \{PTEN_0\}$
$\ell_2 = \{mTORC1_0\}$

$\ell_5 = \{mTORC1_1, PI3K_0, FOXO_0\}$
$\ell_6 = \{PI3K_1\}$
$\ell_7 = \{FOXO_1\}$

$\ell_{11} = \{AKT_1, FOXO_0\}$
$\ell_{12} = \{FOXO_0\}$
$\ell_{13} = \{FOXO_1\}$

$\ell_3 = \{PI3K_1\}$
$\ell_4 = \{PI3K_0\}$

$\ell_8 = \{PI3K_1, mTORC2_1\}$
$\ell_9 = \{mTORC2_0\}$
$\ell_{10} = \{PI3K_0\}$

$\ell_{14} = \{AKT_0, mTORC1_1\}$
$\ell_{15} = \{AKT_1\}$
