# Supplementary material for: Formal Modeling of mTOR Associated Biological Regulatory Network Reveals Novel Therapeutic Strategy for the Treatment of Cancer
Source: Front Physiol. 2017 Jun 13;8:416. doi: 10.3389/fphys.2017.00416 (PMC5468443; doi:10.3389/fphys.2017.00416)
Supplement: Supplementary File 1 — Qualitative models with logical parameters (SMBioNet code). [file DataSheet1.ZIP › Supplementary file 2.doc]

\# MODEL 1

\# K_PI3K = 0
\# K_PI3K+MTORC1 = 0
\# K_PI3K+PTEN = 1
\# K_PI3K+MTORC1+PTEN = 1

\# K_MTORC2 = 0
\# K_MTORC2+FOXO = 0
\# K_MTORC2+MTORC1 = 1
\# K_MTORC2+PI3K = 1
\# K_MTORC2+FOXO+MTORC1 = 1
\# K_MTORC2+MTORC1+PI3K = 1
\# K_MTORC2+FOXO+PI3K = 1
\# K_MTORC2+FOXO+MTORC1+PI3K = 1

\# K_AKT = 0
\# K_AKT+MTORC2 = 0
\# K_AKT+PI3K = 0
\# K_AKT+MTORC2+PI3K = 1

\# K_MTORC1 = 0
\# K_MTORC1+AKT = 0
\# K_MTORC1+FOXO = 1
\# K_MTORC1+AKT+FOXO = 1

\# K_FOXO = 0
\# K_FOXO+AKT = 1
\# K_FOXO+MTORC1 = 0
\# K_FOXO+AKT+MTORC1 = 1

\# K_PTEN = 0
\# K_PTEN+PI3K = 1

\# MODEL 2

\# K_PI3K = 0
\# K_PI3K+MTORC1 = 1
\# K_PI3K+PTEN = 1
\# K_PI3K+MTORC1+PTEN = 1

\# K_MTORC2 = 0
\# K_MTORC2+FOXO = 0
\# K_MTORC2+MTORC1 = 1
\# K_MTORC2+PI3K = 1
\# K_MTORC2+FOXO+MTORC1 = 1
\# K_MTORC2+MTORC1+PI3K = 1
\# K_MTORC2+FOXO+PI3K = 1
\# K_MTORC2+FOXO+MTORC1+PI3K = 1

\# K_AKT = 0
\# K_AKT+MTORC2 = 0
\# K_AKT+PI3K = 0
\# K_AKT+MTORC2+PI3K = 1

\# K_MTORC1 = 0
\# K_MTORC1+AKT = 0
\# K_MTORC1+FOXO = 1
\# K_MTORC1+AKT+FOXO = 1

\# K_FOXO = 0
\# K_FOXO+AKT = 1
\# K_FOXO+MTORC1 = 0
\# K_FOXO+AKT+MTORC1 = 1

\# K_PTEN = 0
\# K_PTEN+PI3K = 1

\# MODEL 3

\# K_PI3K = 0
\# K_PI3K+MTORC1 = 0
\# K_PI3K+PTEN = 1
\# K_PI3K+MTORC1+PTEN = 1

\# K_MTORC2 = 0
\# K_MTORC2+FOXO = 1
\# K_MTORC2+MTORC1 = 1
\# K_MTORC2+PI3K = 1
\# K_MTORC2+FOXO+MTORC1 = 1
\# K_MTORC2+MTORC1+PI3K = 1
\# K_MTORC2+FOXO+PI3K = 1
\# K_MTORC2+FOXO+MTORC1+PI3K = 1

\# K_AKT = 0
\# K_AKT+MTORC2 = 0
\# K_AKT+PI3K = 0
\# K_AKT+MTORC2+PI3K = 1

\# K_MTORC1 = 0
\# K_MTORC1+AKT = 0
\# K_MTORC1+FOXO = 1
\# K_MTORC1+AKT+FOXO = 1

\# K_FOXO = 0
\# K_FOXO+AKT = 1
\# K_FOXO+MTORC1 = 0
\# K_FOXO+AKT+MTORC1 = 1

\# K_PTEN = 0
\# K_PTEN+PI3K = 1

\# MODEL 4

\# K_PI3K = 0
\# K_PI3K+MTORC1 = 1
\# K_PI3K+PTEN = 1
\# K_PI3K+MTORC1+PTEN = 1

\# K_MTORC2 = 0
\# K_MTORC2+FOXO = 1
\# K_MTORC2+MTORC1 = 1
\# K_MTORC2+PI3K = 1
\# K_MTORC2+FOXO+MTORC1 = 1
\# K_MTORC2+MTORC1+PI3K = 1
\# K_MTORC2+FOXO+PI3K = 1
\# K_MTORC2+FOXO+MTORC1+PI3K = 1

\# K_AKT = 0
\# K_AKT+MTORC2 = 0
\# K_AKT+PI3K = 0
\# K_AKT+MTORC2+PI3K = 1

\# K_MTORC1 = 0
\# K_MTORC1+AKT = 0
\# K_MTORC1+FOXO = 1
\# K_MTORC1+AKT+FOXO = 1

\# K_FOXO = 0
\# K_FOXO+AKT = 1
\# K_FOXO+MTORC1 = 0
\# K_FOXO+AKT+MTORC1 = 1

\# K_PTEN = 0
\# K_PTEN+PI3K = 1

\# MODEL 5

\# K_PI3K = 0
\# K_PI3K+MTORC1 = 0
\# K_PI3K+PTEN = 1
\# K_PI3K+MTORC1+PTEN = 1

\# K_MTORC2 = 0
\# K_MTORC2+FOXO = 0
\# K_MTORC2+MTORC1 = 1
\# K_MTORC2+PI3K = 1
\# K_MTORC2+FOXO+MTORC1 = 1
\# K_MTORC2+MTORC1+PI3K = 1
\# K_MTORC2+FOXO+PI3K = 1
\# K_MTORC2+FOXO+MTORC1+PI3K = 1

\# K_AKT = 0
\# K_AKT+MTORC2 = 0
\# K_AKT+PI3K = 0
\# K_AKT+MTORC2+PI3K = 1

\# K_MTORC1 = 0
\# K_MTORC1+AKT = 1
\# K_MTORC1+FOXO = 1
\# K_MTORC1+AKT+FOXO = 1

\# K_FOXO = 0
\# K_FOXO+AKT = 1
\# K_FOXO+MTORC1 = 0
\# K_FOXO+AKT+MTORC1 = 1

\# K_PTEN = 0
\# K_PTEN+PI3K = 1

\# MODEL 6

\# K_PI3K = 0
\# K_PI3K+MTORC1 = 1
\# K_PI3K+PTEN = 1
\# K_PI3K+MTORC1+PTEN = 1

\# K_MTORC2 = 0
\# K_MTORC2+FOXO = 0
\# K_MTORC2+MTORC1 = 1
\# K_MTORC2+PI3K = 1
\# K_MTORC2+FOXO+MTORC1 = 1
\# K_MTORC2+MTORC1+PI3K = 1
\# K_MTORC2+FOXO+PI3K = 1
\# K_MTORC2+FOXO+MTORC1+PI3K = 1

\# K_AKT = 0
\# K_AKT+MTORC2 = 0
\# K_AKT+PI3K = 0
\# K_AKT+MTORC2+PI3K = 1

\# K_MTORC1 = 0
\# K_MTORC1+AKT = 1
\# K_MTORC1+FOXO = 1
\# K_MTORC1+AKT+FOXO = 1

\# K_FOXO = 0
\# K_FOXO+AKT = 1
\# K_FOXO+MTORC1 = 0
\# K_FOXO+AKT+MTORC1 = 1

\# K_PTEN = 0
\# K_PTEN+PI3K = 1

\# MODEL 7

\# K_PI3K = 0
\# K_PI3K+MTORC1 = 0
\# K_PI3K+PTEN = 1
\# K_PI3K+MTORC1+PTEN = 1

\# K_MTORC2 = 0
\# K_MTORC2+FOXO = 1
\# K_MTORC2+MTORC1 = 1
\# K_MTORC2+PI3K = 1
\# K_MTORC2+FOXO+MTORC1 = 1
\# K_MTORC2+MTORC1+PI3K = 1
\# K_MTORC2+FOXO+PI3K = 1
\# K_MTORC2+FOXO+MTORC1+PI3K = 1

\# K_AKT = 0
\# K_AKT+MTORC2 = 0
\# K_AKT+PI3K = 0
\# K_AKT+MTORC2+PI3K = 1

\# K_MTORC1 = 0
\# K_MTORC1+AKT = 1
\# K_MTORC1+FOXO = 1
\# K_MTORC1+AKT+FOXO = 1

\# K_FOXO = 0
\# K_FOXO+AKT = 1
\# K_FOXO+MTORC1 = 0
\# K_FOXO+AKT+MTORC1 = 1

\# K_PTEN = 0
\# K_PTEN+PI3K = 1

\# MODEL 8

\# K_PI3K = 0
\# K_PI3K+MTORC1 = 1
\# K_PI3K+PTEN = 1
\# K_PI3K+MTORC1+PTEN = 1

\# K_MTORC2 = 0
\# K_MTORC2+FOXO = 1
\# K_MTORC2+MTORC1 = 1
\# K_MTORC2+PI3K = 1
\# K_MTORC2+FOXO+MTORC1 = 1
\# K_MTORC2+MTORC1+PI3K = 1
\# K_MTORC2+FOXO+PI3K = 1
\# K_MTORC2+FOXO+MTORC1+PI3K = 1

\# K_AKT = 0
\# K_AKT+MTORC2 = 0
\# K_AKT+PI3K = 0
\# K_AKT+MTORC2+PI3K = 1

\# K_MTORC1 = 0
\# K_MTORC1+AKT = 1
\# K_MTORC1+FOXO = 1
\# K_MTORC1+AKT+FOXO = 1

\# K_FOXO = 0
\# K_FOXO+AKT = 1
\# K_FOXO+MTORC1 = 0
\# K_FOXO+AKT+MTORC1 = 1

\# K_PTEN = 0
\# K_PTEN+PI3K = 1

\# SELECTED MODELS / CHECKED MODELS = 8 / 40 (474ms)
